# Establishing a Self-sustaining Emergency Medicine Point-of-Care Ultrasound Curriculum in an Academic Teaching Hospital in Ethiopia

**DOI:** 10.4314/ejhs.v32i3.8

**Published:** 2022-05

**Authors:** Anne Aspler, Fraser Kegel, Temesgen Beyene, Tigist Zewdu, Berhanu Tesfaye, Alexandra McKnight, Eileen Cheung, Jennifer Bryan, Claire Acton

**Affiliations:** 1 Division of Emergency Medicine, Department of Medicine, Faculty of Medicine, University of Toronto, Toronto, Ontario, Canada; 2 Department of Emergency Medicine, College of Health Sciences, Addis Ababa University, Addis Ababa, Ethiopia; 3 Division of Emergency Medicine, Department of Family and Community Medicine, University of Toronto, Toronto, ON, Canada; 4 Department of Medicine, Cumming School of Medicine, University of Calgary, Alberta, Canada; 5 Toronto Addis Ababa Academic Collaboration in Emergency Medicine (TAAAC-EM), Toronto, ON, Canada

**Keywords:** Point-of-care ultrasound, emergency medicine, residency curriculum, Ethiopia, low- and middle-income countries, educational innovation

## Abstract

**Background:**

Point-of-care ultrasound (POCUS) training has become a standard component of Canadian emergency medicine (EM) residency programs. In resource-limited contexts, including Ethiopia, there is a critical shortage of local clinicians who can perform and teach POCUS. Our aim was to establish an introductory POCUS rotation within the EM residency program at Addis Ababa University (AAU) through The Toronto Addis Ababa Academic Collaboration in Emergency Medicine (TAAAC-EM).

**Methods:**

Through stakeholder engagement, the authors completed a quality improvement initiative and conducted a survey of AAU EM faculty and residents to understand which POCUS scans should be included in a core residency POCUS curriculum, “POCUS1”.

**Results:**

17 residents completed the POCUS1 program and 16 residents completed the written survey. Focused assessment with sonography for trauma, inferior vena cava, and lung (pneumothorax, pleural effusions, and interstitial syndrome) were identified as core introductory topics. Seventeen residents completed the initial POCUS1 program. Three program graduates were supported to become “POCUS1 Master Instructors” to continue the program during the SARS-CoV-2 global pandemic.

**Conclusion:**

The authors identified the highest yield POCUS scans through a written survey, successfully introduced a sustainable core POCUS curriculum at AAU for EM residents, and graduated three master instructors for curriculum continuation. We outline the structure and materials for implementation of POCUS programs for EM trainees and staff in similar low- and middle-income countries.

## Introduction

Point-of care ultrasound (POCUS) training has become a standard component of Canadian emergency medicine (EM) residency training programs. In resource-limited contexts, where formal imaging may be unavailable or unaffordable for patients, availability and clinical application of POCUS training is of even greater importance.

In Addis Ababa, Ethiopia, there is a scarcity of radiologists serving the 10 public hospitals and population of approximately 5 million people (1). At Tikur Anbessa Specialized Hospital (TASH), the primary teaching hospital at Addis Ababa University (AAU), radiology services are not available in the emergency department (ED) afterhours, there is delayed turnaround time for standard xrays, multiple x-ray views are not always available, and there is not consistent access to a computed tomography scanner (2).

Compounding this need is a critical shortage of local clinicians who can perform and teach POCUS. AAU is a well-established academic institution, with multiple educational partnerships aimed at capacity-building. The Toronto Addis Ababa Academic Collaboration in Emergency Medicine (TAAAC-EM) began in 2010 and supports local faculty in providing a three-year EM residency training program. Within this context, we sought to establish an introductory POCUS rotation that could be sustainably run by local faculty (3).

The development of EM POCUS services in low- and middle-income countries (LMICs) requires understanding the local context and balancing the need for diagnostic testing with the capacity to provide high quality training to EM residents (4). Other sub-Saharan Africa POCUS training initiatives discussed in the literature have been mainly limited to under two weeks and rely on visiting teachers to deliver the curriculum (Table 1). There is an increased movement towards sustainability of curriculum delivery, but limited data describing methods to achieve this in settings with few local POCUS experts (4–7).

**Table 1:** Published POCUS Training Programs in Sub-Saharan African Countries

Study	Location, Year	POCUS Curriculum Topics	Training Length	Instructors: Local vs Visiting	Audience
Jones (11)	Kenya, 2020	eFAST, cardiac, lung, DVT, OBGYN, renal, liver, spleen, aorta, soft tissue	3 days	Visiting	39 family medicine physicians and registrars
Henwood (6)	Rwanda, 2017	Cardiac, abdominal, lung, OBGYN, spleen, liver, biliary, renal, soft tissue	10 days	Local and visiting	15 staff physicians
Denny (12)	Tanzania, 2018	FAST, cardiac, abdominal, MSK, Lung, OBGYN	4 weeks	Visiting	229 students, practicing healthcare professionals, school employees
Tafoya (13)	Ghana, 2017	Lung, cardiac, IVC, abdominal, aorta, DVT	10 days	Visiting	18 EM residents 2 EM staff physicians
Shaffer (14)	Tanzania, 2017	FAST, cardiac, lung	5 days	Local and visiting	8 registrar physicians
Reynolds (15)	Tanzania, 2016	FAST, lung, cardiac, gallbladder, aorta, OBGYN	16 days	Visiting	14 registrar physicians
Bell (16)	Kenya, 2016	FAST, lung, OBGYN	3 days	Visiting	81 clinical officers, doctors, nurses, radiographers, lab technicians
Stolz (7)	Uganda, 2015	FAST, cardiac, gallbladder, renal, soft tissue, lung, aorta, IVC, OBGYN, abdominal, procedural, DVT	Integrated 2- year program	Local and visiting	Nurses
Douglass (5)	Rwanda 2013	Not recorded	10 days	Not recorded	10 staff physicians
Adler (17)	Tanzania, 2008	FAST, aorta, hepatobiliary, OBGYN, procedural, soft tissue, renal	4 days	Visiting	4 physicians, 6 clinical officers
Shah (18)	Rwanda, 2015	OBGYN, abdominal, renal, hepatobiliary, cardiac, pleural, vascular, procedural	9 weeks	Visiting	Staff physicians

eFAST: extended focused assessment with sonography in trauma, DVT: deep venous thrombosis, OBGYN: obstetrical and gynecological, MSK: musculocutaneous, IVC: inferior vena cava, EM: emergency medicine, FAST: focused assessment with sonography in trauma

The authors sought to determine which EM POCUS scans are of the highest clinical benefit in the local context through stakeholder engagement and a written survey of faculty and residents and introduce a sustainable basic POCUS curriculum which could be sustained by academic faculty at the TASH ED at AAU.

## Materials and Methods

This was a quality improvement initiative conducted as part of the regular curriculum development process that took place at the TASH ED at AAU through six one-month teaching trips from visiting University of Toronto (UofT) faculty and residents from the Toronto Addis Ababa Academic Collaboration in Emergency Medicine (TAAAC-EM) from February 2018 to February 2020.

Our curriculum design process was inspired by Kern's six step approach (8): 1) Identification of need for POCUS EM skills, 2) Needs assessment, 3) Identification and refinement of goals, 4) Design and introduction of a junior POCUS curriculum, 5) Design and introduction of a Master Instructor (MI) program to promote sustainability, 6) Continuous evaluation and feedback.

In order to create appropriate skills-based objectives, we conducted a written survey of AAU EM faculty and residents to determine which POCUS scans should be prioritized in a PGY (post-graduate year) 1 POCUS rotation (Appendix 1). POCUS scans addressed in the survey included: focused assessment with sonography in trauma (FAST), inferior vena cava (IVC), cardiac, lung (pneumothorax, pleural effusion, interstitial syndrome), gallbladder, abdominal aorta, advanced abdominal (appendicitis, small bowel obstruction), renal and bladder, first trimester pregnancy, deep venous thrombosis (DVT), ocular, musculoskeletal, endotracheal tube placement confirmation, and procedural (nerve blocks, thoracentesis, paracentesis, pericardiocentesis, lumbar puncture). After the results of the survey were analyzed, three scans were selected for the POCUS1 curriculum. All participants completed a written pre-scanning quiz, performed in-person supervised scans for each selected application, and completed a supervised visual and written exam. Highly performing residents were selected through an application process to become master instructors to sustain the POCUS1 curriculum. As this was a quality improvement initiative and not research governed by the research and ethics board, REB approval was not indicated.

## Results

Seven PGY1 residents, ten PGY2 residents, and seven EM faculty participated in this study. Sixteen (94%) residents and three (43%) EM faculty completed the survey. The most requested scans from residents were FAST, cardiac, lung, DVT, ocular, and nerve block scans. Faculty members' most requested scans were advanced cardiac and endotracheal tube confirmation scans.

After discussions with faculty at AAU, we narrowed the focus to (a) topics with greatest relevance to the TASH ED population, and (b) topics which could be feasibly taught by local POCUS-trained faculty: FAST, IVC, and lung.

The POCUS1 rotation includes a flipped classroom model reviewing materials on image generation and interpretation. To be eligible to participate in in-person scanning, residents are required to pass a pre-scanning quiz with a score of 70% (Appendix 2). Then, each resident is required to perform 25 in-person supervised scans for each application and pass a supervised visual and written exam with a score of 100%. Educational materials and scanning techniques were based on the Canadian Emergency Department Echo (EDE) program (9).

Throughout the study period, all 17 residents completed the POCUS1 program. The next step was introduction of a POCUS1 Master Instructor (MI) certification. The SARS-CoV-2 pandemic disrupted travel for in-person teaching by UofT faculty and the need for local instructors became more urgent. Three POCUS-trained faculty were practicing at TASH, including two who were pursuing advanced ultrasound training through the Ultrasound Leadership Academy. Through an application process, three residents were selected by POCUS-trained AAU faculty to become MI candidates.

MI candidates underwent a virtual orientation co-taught by AAU and UofT POCUS faculty. MI candidates supervised junior resident scanning. They were supervised in real-time and provided monitoring and feedback by AAU faculty. MI candidates were provided with a series of POCUS images and descriptive techniques on their mobile devices for use while teaching junior residents at the bedside (Appendix 3). These prompts were embedded on instructors' smart phones and did not require an internet connection to access.

Junior residents logged scans electronically using the EchoLog (ã2022, Montreal, Canada) application. A total of ten junior residents are currently undergoing the POCUS1 training under the supervision of the MI candidates and POCUS AAU faculty. The program will be continuously refined based on continuous feedback from learners and AAU faculty.

## Discussion

We propose a feasible and sustainable model for establishing a core POCUS rotation embedded in an emergency medicine residency training program in LMICs.

Adapting the Canadian EDE curriculum to the Ethiopian emergency medicine context, we did not include first trimester obstetrics and aortic scans in the POCUS1 curriculum. Obstetric patients in their first trimester bypass the TASH ED to attend a dedicated obstetrical emergency intake area run by obstetricians. Given that some graduates will practice outside of TASH, it may be important to introduce first-trimester obstetric scans in the future. For the pragmatic purpose of establishing the POCUS1 program, it was not feasible to have trainees perform scanning shifts in both the ED and dedicated obstetrics triage area.

The abdominal aortic aneurysm (AAA) scan which is included in the Canadian EDE curriculum was also excluded from the POCUS1 curriculum. The mean age of patients undergoing ED POCUS in Addis Ababa was previously estimated to be 35 years, making the prevalence of AAA pathology less relevant in the Ethiopian clinical context (3). The limited availability of surgical interventions in cases of abdominal aortic pathology also make the clinical applicability of the aortic scan questionable, similar to the South African experience (10).

Although not typically included in Canadian introductory POCUS curricula, IVC and advanced lung scanning were considered essential to the POCUS1 rotation. IVC was felt to be high yield in the TASH ED where patients present frequently with hypovolemic or cardiogenic shock. Additionally, many patients may be boarded in the ED for several days due to limited hospital capacity, resulting in Ethiopian EM physicians providing substantial amounts of ongoing critical care. Serial IVC and lung scans assessing volume status have been previously identified as amongst the most clinically relevant scans in an Ethiopian context (3).

Through TAAAC-EM we were able to establish an introductory POCUS curriculum and a Master Instructor program to create a sustainable initiative for POCUS training within an Ethiopian EM residency training program.

While the POCUS1 and MI programs have been successful, we are mindful of the challenges of maintaining a POCUS curriculum. The largest barrier to self-sustaining programming is training a critical mass of local POCUS experts who can independently coordinate rotations over the long-term. Faculty at academic institutions in Ethiopia frequently balance multiple leadership roles with limited support, which makes teaching time-intensive image generation skills an organizational challenge. In retrospect, our initial curriculum model did not explicitly have AAU faculty leading the bedside scanning shifts. In the future, we plan to have UofT faculty provide a mentorship role now that we have a critical mass of trained POCUS1 certified practitioners.

The second largest barrier to self-sustaining programming is the availability of functioning hardware. During the creation of our MI program, the ultrasound machine in the ED was only intermittently available, making it challenging to schedule scanning shifts. Engagement with the decision makers for ongoing support of POCUS infrastructure in the context of emergency residency training programs remains critical to the success of any academic POCUS program in LMICs.

This study should be interpreted in light of several limitations. The POCUS1 curriculum scans are based off survey results from 16 residents and 3 staff and may not represent the highest yield scans for the broader emergency medicine community in Ethiopia, or in other LMICs. As well, the authors are participating in ongoing analysis of the POCUS1 and MI programs, and the sustainability of this program is not yet understood.

Our future plans include introduction of an advanced POCUS rotation, continuing medical education opportunities for faculty, and establishing AAU as a POCUS center of excellence in East Africa. We will also be conducting regular program assessments and modifying curricula with local ultrasound leads in response to local needs.
